# Supplementary material for: The impact of light and thioredoxins on the plant thiol-disulfide proteome
Source: Plant Physiol. 2024 Jan 12;195(2):1536–60. doi: 10.1093/plphys/kiad669 (PMC11142374; doi:10.1093/plphys/kiad669)
Supplement: kiad669_Supplementary_Data [file kiad669_supplementary_data.zip › Supplemental Figures_resubmission_revision2.pdf]

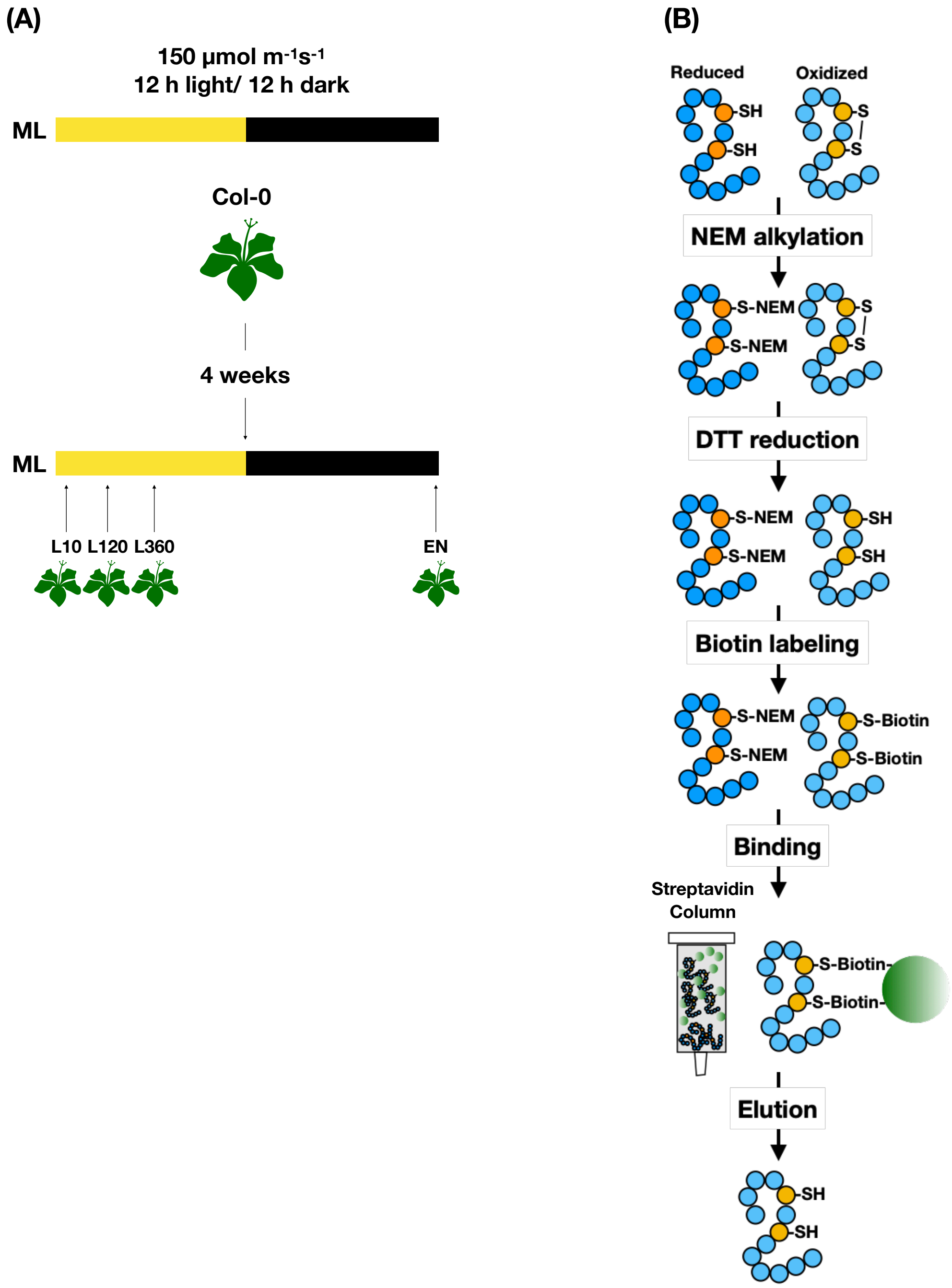

Supplemental Figure S1. The procedures of samples preparation and redox proteomics. (A) Arabidopsis plants were grown under light intensity of 150 µmol photons m^{-2} s^{-1} with a 12-h-dark/12-h-light regime for 4 weeks. To monitor the time course in the light, whole rosette leaves were sampled by shock-freezing in liquid nitrogen at end of night (EN) and 10, 120 and 360 min into the photoperiod. (B) The leaf samples were used for protein extraction in the presence of N-ethylmaleimide (NEM) to block the free thiol residues followed by DTT treatment to reduce the oxidized thiols. The released thiols were labeled with redox-active biotin, and the protein extract was subject to affinity purification using a streptavidin column. The bound proteins were eluted via incubating with DTT.

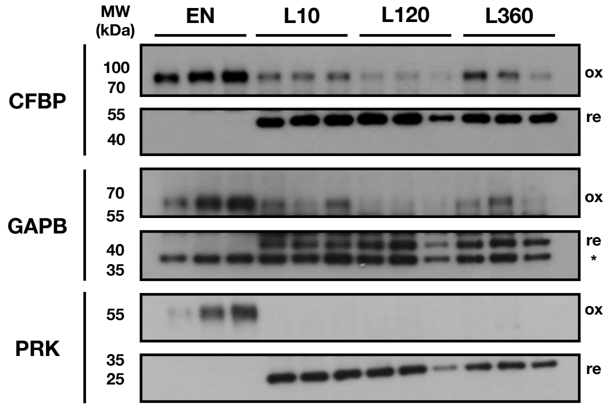

Supplemental Figure S2. Validation of the redox proteomics results by analyzing oxidized and reduced forms of CBC enzymes using protein electrophoretic mobility shift assays as an independent method. The protein oxidation percentages of chloroplastic fructose 1,6-bisphosphatase (CFBP), glyceraldehyde-3-phosphate dehydrogenase B (GAPB) and phosphoribulokinase (PRK) were analyzed via protein electrophoretic mobility shift assay as an independent method. For three independent biological replicates, the reduced thiols of proteins were alkylated using NEM, and the oxidized thiols of proteins were released by treating with DTT. The released thiols were further labeled with methoxypolyethlene glycol maleimide, which resulted in an increase of protein mass of the oxidized form that became distinguishable from the reduced form during gel electrophoresis. The oxidized and reduced forms of the proteins are indicated on the immunoblots. The immunosignals marked “ox” and “re” represent the oxidized and reduced forms of proteins, respectively, while those marked with asterisk represent the signals of non-specific binding. The scanned blots were used to calculate oxidation percentages of CFBP, GAPB and PRK shown in Figure 3.

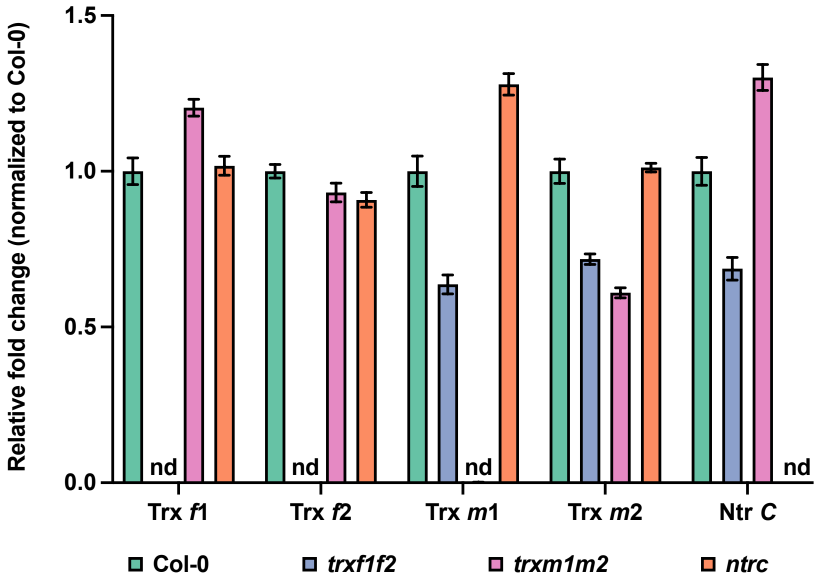

Supplemental Figure S3. Molecular characterization of *trxf1f2*, *trxm1m2* and *ntrc* mutant lines. The expression levels of Trx *f*1, *f*2, *m*1, *m*2 and Ntr *C* were detected using RT-qPCR with gene-specific primers. The gene expression was quantified using $2^{-\Delta\Delta Ct}$ method. The symbol "nd" indicates "not detected". Results are the mean ± SE from six biological replicates.

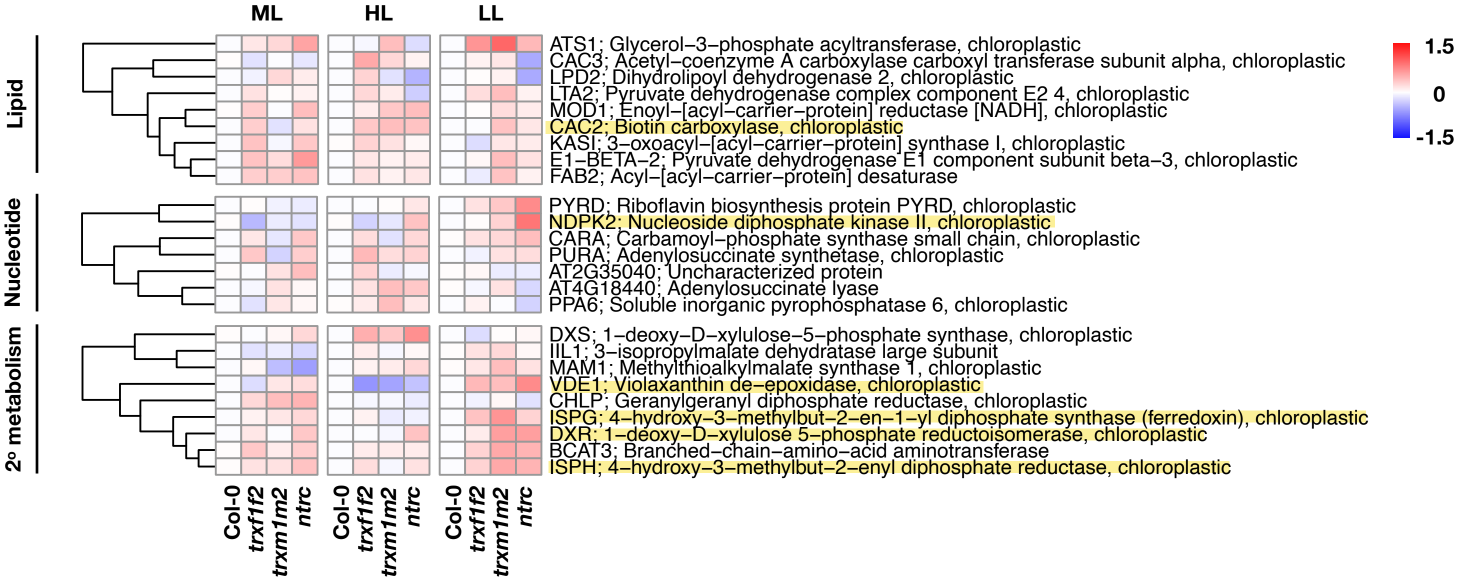

Supplemental Figure S4. Protein oxidation changes in lipid, nucleotide and secondary metabolism across *trxf1f2*, *trxm1m2* and *ntrc* mutants in constant and fluctuating light. Heatmaps summarize the log_2 fold changes of protein oxidation levels in Arabidopsis *trxf1f2*, *trxm1m2* or *ntrc* mutants relative to the wild type (Col-0). The targets with yellow background are proposed to be redox-regulated proteins according to previous studies (Lindahl and Kieselbach, 2009). Proteins were allocated to different groups of biological functions including lipid, nucleotide and secondary metabolism. Plants were grown in the same conditions as indicated in the legend of Figure 4. Data are the means of three to six biological replicates. ML, medium light; FL, fluctuating light; HL, high-light phase of FL; LL, low-light phase of FL; red = increased oxidation, blue = decreased oxidation. Raw data and statistics, see Supplemental Table S4.

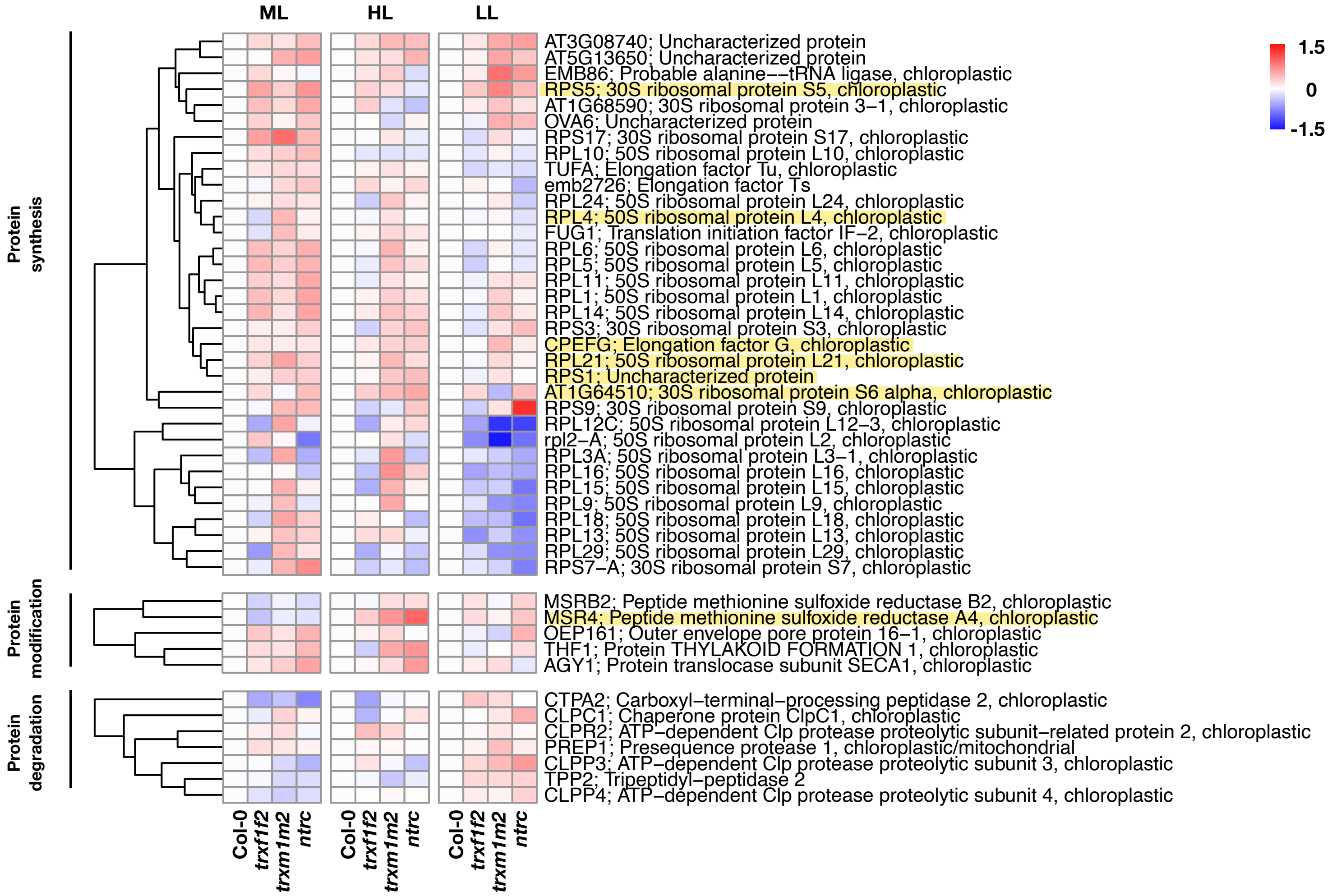

Supplemental Figure S5. Protein oxidation changes in protein metabolism across *trxf1f2*, *trxm1m2* and *ntrc* mutants in constant and fluctuating light. Heatmaps summarize the log_2 fold changes of protein oxidation levels in Arabidopsis *trxf1f2*, *trxm1m2* or *ntrc* mutants relative to the wild type (Col-0). The targets with yellow background are proposed to be redox-regulated proteins according to previous studies (Lindahl and Kieselbach, 2009). Proteins were allocated to different groups of biological functions including protein synthesis, modification and degradation. Plants were grown in the same conditions as indicated in the legend of Figure 4. Data are the means of three to six biological replicates. ML, medium light; FL, fluctuating light; HL, high-light phase of FL; LL, low-light phase of FL; red = increased oxidation, blue = decreased oxidation. Raw data and statistics, see Supplemental Table S4.
